# Correction: Effect of perchlorate on biocementation capable bacteria and Martian bricks

**DOI:** 10.1371/journal.pone.0344195

**Published:** 2026-03-02

**Authors:** Swati Dubey, Shubhanshu Shukla, Nitin Gupta, Rashmi Dikshit, Punyasloke Bhadury, Aloke Kumar

The fourth author’s name is spelled incorrectly. The correct name is: Rashmi Dikshit. The correct citation is: Dubey S, Shukla S, Gupta N, Dikshit R, Bhadury P, Kumar A (2026) Effect of perchlorate on biocementation capable bacteria and Martian bricks. PLoS One 21(1): e0340252. https://doi.org/10.1371/journal.pone.0340252.

An additional affiliation is missing for the fourth author. Rashmi Dikshit is also affiliated with the MATI Carbon India Pvt. Ltd. Bangalore, India.
