# Supplementary material for: Effect of perchlorate on biocementation capable bacteria and Martian bricks
Source: PLoS One. 2026 Jan 29;21(1):e0340252. doi: 10.1371/journal.pone.0340252 (PMC12854443; doi:10.1371/journal.pone.0340252)
Supplement: S1 File — (PDF) [file pone.0340252.s001.pdf]

## Supplementary Methods

- **Biochemical Characterization of the SI_IISc_isolate:**

The characterization of SI_IISc_isolate bacteria was done through a series of biochemical tests using Bacillus Identification kit (HiMedia, India). The results of all biochemical tests are summarized in the table S1.

- **Effect of Perchlorate on Bacterial growth & viability**

To understand the impact of perchlorate on the bacterial growth, we determined the Minimum Inhibitory Concentration of perchlorate for the bacteria through plate assay and broth tests. To understand the effect of varying concentrations of perchlorate on the viability of our bacteria, we also performed live/dead staining using Live/Dead BacLight Kit (ThermoFischer Scientific), performed after 8 hours of incubation in nutrient media+ 2% urea (NBU)with different concentrations of perchlorate along with a control (with no perchlorate).

**(i) Minimum Inhibitory Concentration of Perchlorate**

To know the effect of perchlorate on bacteria, we first determined the minimum inhibitory concentration of perchlorate i.e. the concentration at which bacterial growth is inhibited. This was done by growth curve (quantitative)(refer S3 Fig) & plate assay (qualitative)(refer S4 Fig)

**(ii) Live/Dead Staining**

To understand the effect of perchlorate on viability of bacterial cells, live-dead bacterial staining was performed using Live/Dead BacLight Kit (ThermoFischer Scientific., The bacteria was grown in Nutrient Broth + 2% Urea (NBU media) & the cultures set up were (a) Control (b) SI+ 0.5% Perchlorate (c) SI + 1% Perchlorate (d) SI+2% Perchlorate.

The kit protocol was followed for the preparation of slides and were observed after 8 hours of incubation, and observed at 63X under fluorescence microscope using appropriate fluorescence filters.
