# Supplementary material for: Effect of perchlorate on biocementation capable bacteria and Martian bricks
Source: PLoS One. 2026 Jan 29;21(1):e0340252. doi: 10.1371/journal.pone.0340252 (PMC12854443; doi:10.1371/journal.pone.0340252)
Supplement: S1 Table — (DOCX) [file pone.0340252.s002.docx]

***S1 Table. Summary of Biochemical tests done for SI_IISc_isolate bacteria.***

| **SR. No.** | **Biochemical Tests** | **Positive/Negative** |
| --- | --- | --- |
| **1** | Malonate Utilization | N |
| **2** | Methyl Red-Vogeus Proskaeur Test or Acetoin Production | N |
| **3** | Citrate Utilization | P |
| **4** | Beta Galactosidase or ONPG test | N |
| **5** | Nitrate Utilization | N |
| **6** | Catalase | P |
| **7** | Arginine Utilization | P |
| **8** | Sucrose Utilization | N |
| **9** | Mannitol Utilization | N |
| **10** | Glucose Utilization | N |
| **11** | Arabinose Utilization | N |
| **12** | Trehalose Utilization | N |
| **13** | Urea hydrolysis | P |
| **14** | Gelatin Hydrolysis | N |
| **15** | Oxidase | N |
