# Supplementary material for: Effect of perchlorate on biocementation capable bacteria and Martian bricks
Source: PLoS One. 2026 Jan 29;21(1):e0340252. doi: 10.1371/journal.pone.0340252 (PMC12854443; doi:10.1371/journal.pone.0340252)
Supplement: S2 Table — The data analysis was done through X’Pert HighScore Plus Pattern Software. (DOCX) [file pone.0340252.s005.docx]

**S2 Table. 2Theta values and d-spacing as matched by ICSD database (reference IDs given) through X-Ray Diffraction study of the precipitate made by SI through its ureolysis activity. The data analysis was done through X’Pert HighScore Plus Pattern Software.**

| **No.** | **Pos. [°2Th.]** | **d-spacing [Å]** | **Rel. Int. [%]** | **Identified phase** | **hkl** | **ICSD Reference IDs** |
| --- | --- | --- | --- | --- | --- | --- |
| 1 | 23.12748 | 3.8427 | 9.69 | calcite | 012 | 98-004-0544 |
| 2 | 29.49984 | 3.02552 | 100 | calcite | 104 | 98-004-0544 |
| 3 | 35.99091 | 2.49335 | 10.87 | vaterite | 024 | 98-001-8127 |
| 4 | 39.45488 | 2.28206 | 16.38 | calcite | 113 | 98-004-0544 |
| 5 | 43.1715 | 2.09381 | 13.87 | vaterite | 026 | 98-001-8127 |
| 6 | 47.62101 | 1.90804 | 15.95 | vaterite | 027 | 98-001-8127 |
| 7 | 48.57673 | 1.87271 | 15.99 | calcite | 116 | 98-004-0544 |
| 8 | 57.35916 | 1.60509 | 5.89 | calcite | 122 | 98-004-0544 |
| 9 | 60.95073 | 1.51882 | 1.62 | calcite | 208 | 98-004-0544 |
| 10 | 64.58039 | 1.44195 | 3.49 | aragonite | 122 | 98-016-1819 |
